# Relationship Between Product Features and the Prices of e-Cigarette Devices Sold in Web-Based Vape Shops: Comparison Study Using a Linear Regression Model

**DOI:** 10.2196/49276

**Published:** 2024-05-09

**Authors:** Yanyun He, Qian Yang, Yousef Alish, Shaoying Ma, Zefeng Qiu, Jian Chen, Theodore Wagener, Ce Shang

**Affiliations:** 1 Center for Tobacco Research The Ohio State University Wexner Medical Center Columbus, OH United States; 2 Center for Biostatistics Department of Biomedical Informatics The Ohio State University Columbus, OH United States; 3 Department of Computer Science and Engineering The Ohio State University Columbus, OH United States; 4 Medical Oncology Division Department of Internal Medicine The Ohio State University Columbus, OH United States

**Keywords:** e-cigarette devices, price, web-based vape shop, battery capacity, output wattage, e-cigarette, vape, vaping, consumers, costs, prices, pricing, feature, features, device, devices, consumer, cost, smoking products, product safety, consumer safety, smoking, smoker, smokers, tax, taxes, taxation, economic, economics, regression, regression model, health economics

## Abstract

**Background:**

Open-system electronic cigarette (EC) product features, such as battery capacity, maximum output wattage, and so forth, are major components that drive product costs and may influence use patterns. Moreover, continued innovation and monitoring of product features and prices will provide critical information for designing appropriate taxation policies and product regulations.

**Objective:**

This study will examine how product features are associated with the prices of devices sold in web-based vape shops.

**Methods:**

We draw samples from 5 popular, US-based, web-based vape shops from April to August 2022 to examine starter kits, device-only products, and e-liquid container–only products. We implemented a linear regression model with a store-fixed effect to examine the association between device attributes and prices.

**Results:**

EC starter kits or devices vary significantly by type, with mod prices being much higher than pod and vape pen prices. The prices of mod starter kits were even lower than those of mod devices, suggesting that mod starter kits are discounted in web-based vape shops. The price of mod kits, mod device–only products, and pod kits increased as the battery capacity and output wattage increased. For vape pens, the price was positively associated with the volume size of the e-liquid container. On the other hand, the price of pod kits was positively associated with the number of containers.

**Conclusions:**

A unit-based specific tax, therefore, will impose a higher tax burden on lower-priced devices such as vape pens or pod systems and a lower tax burden on mod devices. A volume- or capacity-based specific tax on devices will impose a higher tax burden on vape pens with a larger container size. Meanwhile, ad valorem taxes pegged to wholesale or retail prices would apply evenly across device types, meaning those with advanced features such as higher battery capacities and output wattage would face higher rates. Therefore, policy makers could manipulate tax rates by device type to discourage the use of certain device products.

## Introduction

The electronic cigarette (EC) market has experienced a surge in growth, especially among adolescents and young adults in the United States, which prompted a series of government regulatory actions such as flavor product restrictions and e-cigarette taxes at various government levels [1]. Although ECs pose addiction risks among adolescents and young adults, they also have the potential to help people who smoke combustible tobacco to quit smoking [2], making EC regulations challenging and controversial. Nonetheless, effective EC regulations require that policy makers weigh the benefits and risks of ECs [3,4].

One important feature distinguishing ECs from cigarettes is the wide range of EC models, ranging from basic disposable models to more complex rechargeable devices, which may affect product appeal, use patterns, and health consequences. The use of certain EC devices such as mods and pods may be associated with more frequent EC use and nicotine dependence symptoms [5-7]. The majority of adolescent and young adult EC users report using rechargeable pods (eg, JUUL) [7-11], while smokers who successfully quit smoking are more likely to use open-tank systems or mods [12,13]. The choices of models are further associated with the frequency of EC use and nicotine dependence, making EC devices or models an important product attribute for policy makers to regulate [5-7].

In addition to regulating product attributes by implementing product standards, policy makers could also impose taxes on ECs, including devices, to change their appeal. To attract new users, many EC retailers offer starter kits at discounted prices [14,15], which typically include a rechargeable device and replaceable e-liquid tanks or pods [16,17]. Therefore, taxes and promotion restrictions may be needed to decrease the affordability or appeal of these starter kits to prevent youth initiation. On the other hand, the perceived cost of the devices may make ECs seem expensive compared to traditional tobacco products, which may prevent people from switching from cigarettes to ECs [18,19]. Therefore, monitoring EC device prices and attributes associated with price differences is crucial for policy makers and public health officials to make informed decisions about EC taxation and other pricing policies [5,6].

Rapid advancements in technology have resulted in an increasing number of EC devices being offered in the market, making it challenging to monitor the market and keep track of pricing trends. The existing surveillance data also have the limitation of only providing information on brands sold in brick-and-mortar stores and missing the products sold in other retail channels such as vape shops and web-based stores. Prior studies examining web-based EC stores or vendors revealed that the prices offered digitally were much cheaper than physical stores and few collected sales taxes in their state of business or based on shipping addresses [20,21]. Web-based retail websites engage in a variety of promotional strategies, such as promo or discounts, customer rewards, loyalty programs, and so forth [20,21]. Moreover, they use multiple marketing techniques such as linking their websites to social media platforms, using celebrity endorsement, posing misleading messages about ECs (eg, ECs are healthier, cleaner, and effective as a cessation aid), and so forth, to attract younger population [22-24]. As the US Food and Drug Administration (FDA) issued denial orders to approximately 1 million EC-flavored products in 2020, how this will shift the marketplace and product features remains unknown. It is therefore critical to conduct comprehensive and rapid surveillance on EC devices.

Finally, a growing literature suggests that EC taxes and prices are effective in reducing EC use, with a 10% increase in prices associated with an 11.5% decrease in sales or purchases [25]. However, it is also shown that the current tax bases for ECs vary significantly by state; some states adopt specific taxes, whereas others adopt ad valorem taxes. Given that ECs have a wide range of configurations and features, different tax bases may lead to different tax burdens on different EC types (eg, vape pens vs disposables) [26]. The taxation policies also differ by state regarding whether to tax devices, which are the more durable components compared to refillable cartridges or e-liquid in open-system ECs. A better understanding of EC devices and their costs is needed to guide taxation policies for devices.

In response to these research needs, this study aims to bridge the gap in the literature by analyzing EC device data from popular web-based retailers to evaluate the distribution of device prices and features and investigate the associations between device characteristics and prices in the marketplace using a hedonic pricing model [27]. The results of this study will provide insights to policy makers considering product standards and taxation policies for ECs.

## Methods

### Data Sources

After conducting extensive research through a combination of Google searches and Reddit discussions in 2021, we curated a list of 5 popular web-based vaping shops. Using the latest information available, we prioritized the top results from Google searches and Reddit threads. Specifically, we focused on the top 3 results without physical addresses from Google and identified 2 highly regarded shops without physical addresses from a Reddit discussion dated in 2020. This comprehensive approach ensured that our selection process was thorough and reflective of the most current and popular web-based vape shops available.

From April to August 2022, we conducted a study on open-system EC device products sold from these 5 web-based vape shops. In total, we identified 1166 reusable products after charging or changing batteries. These products include starter kits, device-only products, and container-only products. Starter kits refer to products that include both the heating device and containers, such as mod kits, pod kits, and vape pens. Device-only products only include the heating devices, such as mod and pod devices. Container-only products are just replacement tanks or pods and contain no liquid or solutions. To examine the relationship between prices and device attributes, we focused our analysis on starter kits and device-only products as replacement tanks or pods are components instead of devices.

### Outcome Variable

The outcome variable in our analysis was the log-transformed effective price (ie, after discounts) in US dollars, extracted from the store web pages.

### Explanatory Variables

The following product attributes were selected for regression analysis: the number of containers (ie, tanks or pods or cartridges), container volume size (ie, maximum e-liquid capacity per tank, pod, or cartridge in mL), maximum output wattage (divided into 3 groups with each group comprising approximately one-third of the total observations: 5-39 W, 40-85 W, and more than 85 W), and battery capacity (divided into 3 groups: less than 900 mAh, 900-1499 mAh, and 1500 mAh and more). For the products with missing battery capacity (n=488) or missing wattage information (n=91), we manually checked each product web page. We found that the missing battery capacity in mod kits (n=273) or mod device–only products (n=209) was due to their use of 18,650- or 21,700 mAh–sized batteries and a lack of inclusion in the kits or devices. The missing battery capacity information on other products (n=6) and missing wattage information were purely due to a lack of information on the product web pages. To fully use the sample that we collected for the analysis, a missing category was added for output wattage and battery capacity. In addition to these attributes, the number of coils, rings, cables, chargers, batteries, glasses, and chips were included as control variables.

### Statistical Methods

We used a 3-step approach to investigate pricing patterns and product attributes of EC devices sold in web-based vape shops. First, we computed the price distribution for all EC device products and identified the respective brands. Second, we examined the battery capacity and maximum output wattage features of products for starter kits and device-only products. Finally, we analyzed a linear regression model to examine the association between device prices and product attributes using a hedonic pricing model [27], controlling for store-specific unobservable factors using store-fixed effect and stratified by device types (mod kit, mod devices only, pod kits, and vape pens). Pod device–only products were excluded from the analysis because of the small sample size (n=8). SEs were clustered at the store level to account for intertemporal correlations among products sold in the same store. As the outcome variable was the log form of device price, our estimates reflect the percentage change in price due to a 1-unit change in a continuous independent variable. In the case of a categorical independent variable, our estimates indicate the percentage change in price for being in a certain category compared to the comparison category.

Given that our sample comprises a large number of brands (93 brands) and many brands (59/93, 63% brands) have fewer than 5 products, we did not control for brand-fixed effects. Nonetheless, we conducted sensitivity analysis and estimated alternative models where brand effects are controlled using random effects and generalized estimating equations.

### Ethical Considerations

In this study, we collected data from 5 US-based, web-based vape shops. Thus, no human subjects were involved, and the determination of no human subjects was approved by the Ohio State University institutional review board (study 2020E1328).

## Results

Table 1 shows the summary statistics of EC device products. We identified 1166 products from 93 unique brands, including 427 mod kits, 348 pod kits, 50 vape pens, 229 mod device–only products, 8 pod device–only products, and 104 replacement tanks or pods. Among starter kits, mod kits have a mean price of US $51.46 (SD US $24.72), which is significantly higher (*P*<.001) compared to pod kits (US $24.72, SD US $8.78) and vape pens (US $29.50, SD US $15.60). The mean price of mod kits is significantly lower (*P*<.001) than that of mod device–only products (US $58.93, SD US $41.98). In addition, the median price of mod starter kits is about US $6 cheaper than the sum of individual mod device and e-liquid container prices (US $44.99 and US $8.99, respectively), suggesting that mod kit prices are heavily discounted.

**Table 1:** Summary statistics of EC^a^ device products from 5 popular, US-based, web-based vape shops from April to August 2022 (n=1166).

Product type			Brands, n		Price (US $), mean (SD)		Price (US $), range		Price (US $), median (IQR)
**Starter kits**									
	Mod kit (n=427)	53		51.46 (24.23)		17.99-244.99		47.99 (36.99-59.99)	
	Pod kit (n=348)	58		24.72 (8.78)		4.99-84.99		24.99 (19.99-29.95)	
	Vape pen (n=50)	16		29.50 (15.60)		11.95-99.95		24.97 (19.99-34.49)	
**Device-only products**									
	Mod device–only products (n=229)	48		58.93 (41.98)		17.99-299.99		44.99 (37.95-59.99)	
	Pod device–only products (n=8)	8		11.73 (6.95)		3.99-20.99		9.97 (5.71-18.49)	
**Container-only products**									
	Replacement tanks or pods (n=104)	19		11.80 (7.83)		1.99-33.99		8.99 (4.99-12.99)	

^a^EC: electronic cigarette.

**Figure 1:**
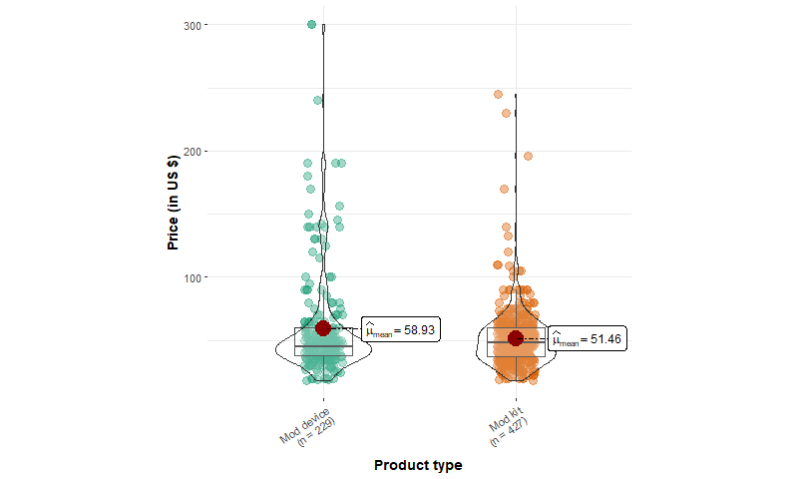
Price distribution of mod products from 5 US-based popular vape shops from April to August 2022 (mod device: n=229, mod kit: n=427).

**Figure 2:**
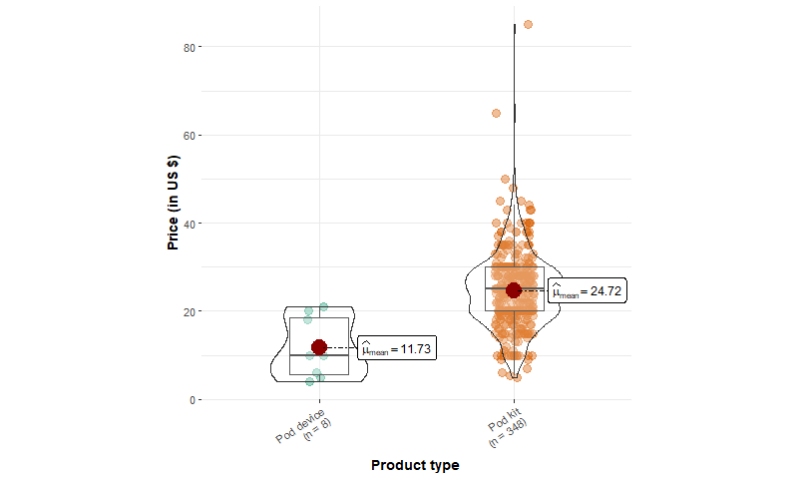
Price distribution of pod products from 5 US-based popular vape shops from April to August 2022 (pod device: n=8, pod kit: n=348).

**Figure 3:**
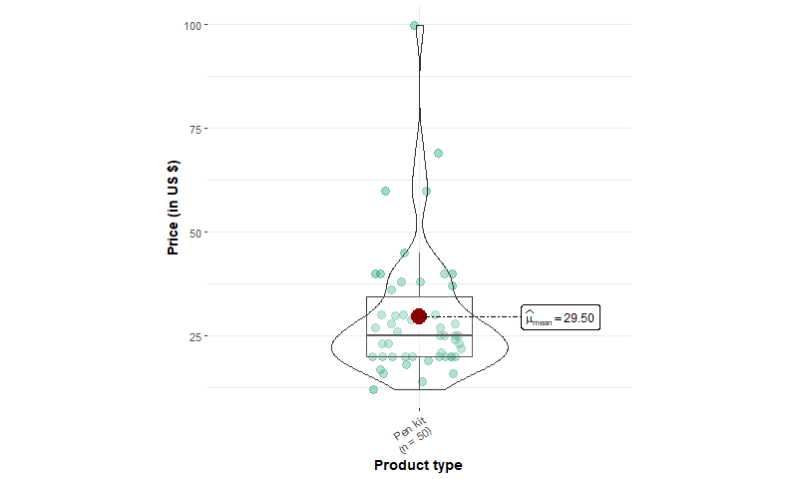
Price distribution of vape pens from 5 US-based popular vape shops from April to August 2022 (vape pen: n=50).

**Figure 4:**
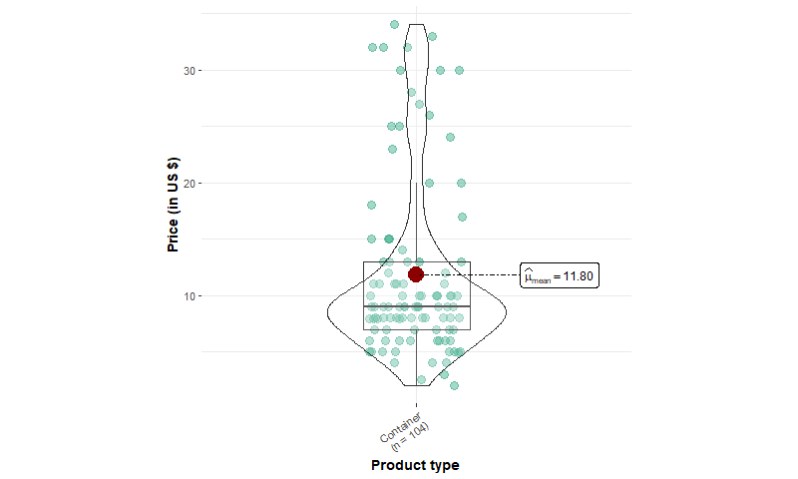
Price distribution of replacement tanks or pods from 5 US-based popular vape shops from April to August 2022 (container: n=104).

The price distribution for all devices except pod kits, which follow a normal distribution, is positively skewed (ie, having high prices; Figures 1-4). Few pod device–only products (n=8) were identified, and they were significantly less expensive than pod kits. This reveals that web-based stores are more likely to sell pod devices in starter kits instead of individual products. The price distributions of vape pens and pod kits are similar: the lower quartile and median prices for vape pens and pod kits are almost identical.

Table 2 displays the distribution of product attributes based on the device type. Pod kits tend to have more replacement tanks or pods included in the kits than mod kits and vape pens. However, the volume size of the containers in mod kits is significantly larger than that in pod kits and vape pens. Most mod kits (396/427, 92.74%) have a battery capacity above 1500 mAh (123/427, 28.81%) or high-end 18,650- and 21,700 mAh–sized batteries (273/427, 63.93%), which are not included in the kits. On the other hand, most pod kits (282/348, 81.04%) have a battery capacity below 900 mAh (152/348, 43.68%) or between 900 mAh and 1500 mAh (130/348, 37.36%). This is probably because these products also typically have much higher concentrations of nicotine so they can still deliver high doses of nicotine with lower wattage and therefore do not need the same battery power. Of the 50 vape pens, 21 (42%), 14 (28%), and 12 (24%) have a battery capacity above 1500 mAh, between 900 mAh and 1500 mAh, and below 900 mAh, respectively. Additionally, 90.63% (387/427) of mod kits have output wattage between 40 W and 85 W (194/427, 45.43%) or above 85 W (193/427, 45.2%), while the majority of pod kits (293/348, 84.2%) have output wattage less than 40 W (216/348, 62.07%) or between 40 W and 85 W (77/348, 22.13%). Most vape pens (21/50, 42%) have output wattage below 40 W. The majority of the mod device–only products (209/229, 91.27%) are sold without including batteries (18,650 or 21,700 mAh) in web-based vape shops. More than half (154/229, 67.25%) of the mod device–only products have output wattage greater than 85 W. These findings suggest that mod kits and mod device–only products are distinct from pod kits and vape pens in terms of battery capacity, output wattage, and volume size.

Table 3 illustrates the association between device prices and product attributes, stratified by device types (mod kit, mod device–only products, pod kits, and vape pens). We found that the price of mod kits is not significantly associated with the number of tanks or pods (*P*=.84) included or with the volume size of the container (*P*=.07). Mod kits with advanced battery sizes (18,650 or 21,700 mAh) are priced 21.5% higher than those with less than 900 mAh batteries, even though the battery is not included in the kit. Mod kits with output wattage over 85 W are priced 27.3% higher than those with less than 40-W output. Similarly, the prices of mod device–only products are higher when they have greater battery capacity or output wattage.

On average, the price of pod kits is 10% higher as the number of tanks or pods increases. However, the price of pod kits is not significantly associated with the volume size of tanks or pods (*P*=.60). Furthermore, the price of pod kits is positively associated with battery capacity, with a capacity between 900 mAh and 1500 mAh and greater than 1500 mAh being 16% and 23.9% higher, respectively, than those with less than 900 mAh battery. The price of pod kits with output wattage between 40 W and 85 W is 12.5% higher compared to those with output wattage less than 40 W.

Unlike mod and pod devices that can be sold as part of a starter kit or as individual devices, vape pens are rechargeable devices sold exclusively as starter kits. The price of vape pens is positively associated with volume sizes, with every 1-mL increase in volume size associated with 3.7% higher prices. In addition, compared to vape pens with a battery capacity below 900 mAh, those with a battery capacity above 1500 mAh are priced 11.9% higher. Output wattage is not significantly associated with the prices of vape pens.

The sensitivity analysis using a random effect model or generalized estimating equations to account for brand effects is reported in Multimedia Appendix 1. The results are very similar to our main findings using ordinary least square regressions.

**Table 2:** The distribution of product features based on product type from 5 popular, US-based, web-based vape shops from April to August 2022 (n=1062).

Product features			Mod kits (n=427)		Pod kits (n=348)		Vape pens (n=50)		Mod device (n=229)		Pod device (n=8)
Tanks or pods, mean (SD)			1.103 (0.327)		1.325 (0.516)		1 (0.000)		N/A^a^		N/A
Tank or pod volume size, mean (SD)			4.695 (1.968)		2.709 (1.162)		3.117 (2.102)^b^		N/A		N/A
**Battery capacity, n (%)**											
	Capacity<900 mAh	5 (1.2)		152 (43.7)		12 (24)		4 (1.8)		8 (100)	
	900 mAh≤capacity<1500 mAh	26 (6.1)		130 (37.4)		14 (28)		3 (1.3)		N/A	
	Capacity≥1500 mAh	123 (28.8)		63 (18.1)		21 (42)		13 (5.7)		N/A	
	Battery not included	273 (63.9)		N/A		N/A		209 (91.3)		N/A	
	Battery missing	N/A		3 (0.9)		3 (6)		N/A		N/A	
**Output wattage, n (%)**											
	Output<40 W	28 (6.6)		216 (62.1)		21 (42)		7 (3.7)		4 (50)	
	40 W≤output<85 W	194 (45.4)		77 (22.1)		10 (20)		54 (23.6)		N/A	
	Output≥85 W	193 (45.2)		2 (0.6)		1 (2)		154 (67.3)		N/A	
	Wattage output missing	12 (2.8)		53 (15.2)		18 (36)		4 (6.1)		4 (50)	

^a^N/A: not applicable.

^b^9 out of 50 vape pens had missing volume size information.

**Table 3:** The association between product features and the prices of electronic cigarette devices stratified by device type from 5 popular, US-based, web-based vape shops from April to August 2022 (n=1045).

Products and features				Coefficient		*P* value
**Mod kits (n=427)**						
	Number of tanks or pods			0.008		.84
	Tank or pod volume size			0.038		.07
	**Battery capacity: <900** **mAh** **as a comparison group**					
		900 mAh≤capacity<1500 mAh	0.117		.27	
		Capacity≥1500 mAh	0.139		.06	
		Battery not included	0.215^a,b^		.03	
	**Maximum output wattage: <40 W as a comparison group**					
		40 W≤output<85 W	0.049		.35	
		Output≥85 W	0.273^a,b^		.02	
		Wattage output missing	–0.098		.55	
**Mod device–only products (n=229)**						
	**Battery capacity: <900** **mAh** **as a comparison group**					
		900 mAh≤capacity<1500 mAh	0.503^a,b^		.03	
		Capacity≥1500 mAh	1.034^a,b^		.04	
		Battery not included	0.844^a,b^		.04	
	**Maximum output wattage: <40 W as a comparison group**					
		40 W≤output<85 W	0.455^b,c^		.01	
		Output≥85 W	0.693^b,c^		.003	
		Wattage output missing	0.532		.07	
**Pod kits (n=348)**						
		Number of tanks or pods	0.100^a,b^		.04	
		Tank or pod volume size	0.011		.60	
	**Battery capacity: <900** **mAh** **as a comparison group**					
		900 mAh≤capacity<1500 mAh	0.160^b,d^		<.001	
		Capacity≥1500 mAh	0.239^b,c^		.008	
		Battery missing^e^	0.506^b,c^		.002	
	**Maximum output wattage: <40 W as a comparison group**					
		40 W≤output<85 W	0.125^a,b^		.05	
		Output≥85 W^e^	–0.023		.67	
		Wattage output missing	0.046		.12	
**Vape pens (n=41)**						
	Vape pen volume size			0.037^b,d^		.001
	**Battery capacity: <900** **mAh** **as a comparison group**					
		900 mAh≤capacity<1500 mAh	0.093		.14	
		Capacity≥1500 mAh	0.119^b,c^		.008	
		Battery missing	0.765^b,d^		.001	
	**Maximum output wattage: <40 W as a comparison group**					
		40 W≤output<85 W	–0.001		.99	
		Output≥85 W^f^	–0.244^b,c^		.008	
		Wattage output missing	–0.193		.06	

^a^*P*<.05.

^b^All regressions were controlled for accessories.

^c^*P*<.01.

^d^*P*<.001.

^e^Since only 2 pod kits have output wattage greater than 85 W and only 3 pod kits have missing battery capacity information, their estimated coefficients have limited statistical relevance.

^f^Since only 1 vape pen has an output wattage greater than 85 W, the estimated coefficient has limited statistical relevance.

## Discussion

Using unique data gathered from 5 web-based stores that sell nationally, this study examines the pricing of rechargeable EC starter kits and devices. The findings reveal that the prices of mod devices and starter kits are on average US $59 to US $51, respectively, approximately twice as high as those of pod starter kits (US $25) and vape pens (US $30). Moreover, all rechargeable devices are much more expensive than disposable devices (cost about US $8) [28]. The average price of mod starter kits (US $51) from the 5 stores is similar to the price reported in a 2016 study (US $56) [20], which evaluated starter kit costs from 44 web-based vendors. Therefore, consumers considering using ECs, especially mod products, can expect relatively high initial costs. This supports previous findings suggesting that consumers tend to choose more affordable disposable options when first experimenting with ECs [29].

Furthermore, we discovered that the price distribution of both mods (starter kits and devices) and vape pens are positively skewed, suggesting that certain products are priced much higher than their average counterparts. This finding is consistent with prior studies based on web-based vendors and studies using the Standardized Tobacco Assessment for Retail Settings: Vape Shops surveillance tool to document prices that found that advanced mod products are priced much higher than regular mods [30,31].

The price analysis of EC devices further illustrates that greater battery capacity and output wattages are associated with higher device or kit prices. This is not surprising given that battery capacity and output wattage are key factors that determine nicotine delivery and user behaviors [21,32,33]. Studies have shown that tank ECs (eg, mod devices) can achieve much higher blood nicotine levels over a longer duration [34]. Survey data also suggest that smokers who successfully quit cigarette smoking using ECs are more likely to use tanks or mod devices [12,13]. It is possible that the higher output wattage and battery capacity, which ensure longer use before needing to be charged and reduce the risk of unexpected power outages, lead to more frequent e-cigarette use and may have assisted in transitions from smoking to vaping. However, greater battery capacity and output wattages could also attract youth and young adult users who report often trying or using multiple devices. Nonetheless, higher output wattage could also expose users to higher toxicant emissions and exposure to higher amounts of particulate matter, which may be harmful to human health [35,36]. The FDA and policy makers may need to take all of the factors (eg, product appeals in youth vs adult smokers) into consideration when setting product standards for batteries and volume sizes.

Our findings provide several key insights about designing EC pricing policies (eg, taxes) for devices. While there is growing literature that increasing EC taxes and prices reduces consumption, there is a lack of evidence that distinguishes between EC types and components, such as devices versus consumables such as e-liquid and cartridges. As a growing number of states start to tax ECs, not all EC-taxing states impose excise taxes on devices. Moreover, there is no clear guidance on how best to tax devices such as choosing tax rates and bases. Our findings suggest that EC starter kits or devices vary significantly by type, with mod prices being much higher than pod and vape pen prices. A unit-based specific tax therefore will impose a higher tax burden on lower-priced devices such as vape pens or pod systems and a lower tax burden on mod devices. A volume- or capacity-based specific tax on devices will impose a higher tax burden on vape pens with large container sizes. On the other hand, ad valorem taxes based on wholesale or retail prices will impose uniform tax burdens across all device types and consequently tax devices with higher battery capacities and output wattage at a higher rate. Therefore, policy makers could manipulate tax rates by device type to discourage the use of certain device products according to the health literature on the relative harms of ECs. For example, if cheap devices are preferred by smokers who are considering ECs, ad valorem tax may be preferred over specific taxes as the former imposed lower taxes on lower-priced products. In contrast, if the goal is to prevent youth from trying ECs and youth are more interested in cheap devices, a specific tax will be more favorable than ad valorem taxes in raising the prices of cheap devices.

In addition to taxation policies, our findings also highlight the importance of promotion restrictions. In web-based stores, the prices of mod starter kits are even lower than those of mod devices, suggesting mod starter kits are discounted. If mod products are mostly used by adult smokers to quit and the initial costs of ECs are a barrier to completely transitioning from cigarettes to ECs, such discounts should be allowed. However, if mods are found to attract youth and young adults, promotion restrictions may be needed to reduce their affordability.

Finally, we used data collected from 5 web-based stores that sell nationally. Although these data are not representative of the US web-based EC marketplace, they provide valuable information on device attributes and costs. It is also important to acknowledge that web-based stores or sales lead to challenges for regulations, including low prices and low compliance with state taxes [21,37]. A prior study further shows that there are international sites that sell ECs to the United States and these sites did not have age verification and detectable health warnings [38]. Future research is needed to understand how international markets in the web-based space may impact use behaviors, price minimization, and policy effectiveness.

There are some limitations of this study. First, we have very limited data on pod devices sold as stand-alone products and therefore do not have sufficient statistical power to conclude the pricing differences between pod starter kits and pod devices. Future studies are needed to address this gap. Second, we did not control for all the factors that affect prices because many factors are either not available or not measurable, such as production costs, consumer preferences, and so forth. Future studies may address this limitation. Nonetheless, we assessed all attributes that are presented on the web-based store web page, which arguably contains all the information that consumers see when they make purchasing decisions. Finally, the US FDA has approved a limited list of EC products. Many e-liquid products have or will become illegal for either failing to submit a premarket approval application or having their applications denied. Therefore, the demand for open-system devices could be significantly reduced as a result. However, given that many e-liquid products remain available in the marketplace and devices could still be used with 0-nicotine e-liquid, we consider monitoring device features continuing to be an important endeavor.

In summary, we provide the first assessment of how product features are associated with device or starter kit prices for the following distinct device types sold in the US digital market: mods, vape pens, and pods. The results can be used to design EC product standards and pricing policies by policy makers.

## Appendix

Multimedia Appendix 1 Sensitivity analysis.

## Abbreviations

EC: electronic cigarette
FDA: Food and Drug Administration

## Data Availability

The data sets generated and analyzed during this study are available from the corresponding author on reasonable request.
