# Supplementary material for: Relationship Between Product Features and the Prices of e-Cigarette Devices Sold in Web-Based Vape Shops: Comparison Study Using a Linear Regression Model
Source: JMIR Form Res. 2024 May 9;8:e49276. doi: 10.2196/49276 (PMC11117130; doi:10.2196/49276)
Supplement: Multimedia Appendix 1 [file formative_v8i1e49276_app1.docx]

**Appendix Table 1: Sensitivity analysis.**

| **Variables** | **GEE** | **Random Effect** |
| --- | --- | --- |
| **Mod kits (n=427)** | | |
| # of tanks/pods | -.004  (.92) | -.007  (.87) |
| Tank/pod volume size | .031***  (.001) | .019*  (.02) |
| **Battery capacity – <900mah as a comparison group** | | |
| 900mah≤capacity<1500mah | .068  (.68) | .006  (.97) |
| Capacity≥1500mah | .106  (.51) | .077  (.62) |
| Battery not included | .186  (.28) | .156  (.31) |
| **Maximum output wattage – <40w as a comparison group** | | |
| 40w≤output<85w | .098  (.26) | .167**  (.01) |
| Output≥85w | .325***  (.001) | .410***  (<.001) |
| Wattage output missing | -.118  (.33) | -.140  (.15) |
| **Mod Device-only (n=229)** | | |
| **Battery capacity – <900mah as a comparison group** | | |
| 900mah≤capacity<1500mah | .343  (.06) | .347  (.42) |
| Capacity≥1500mah | .683**  (.008) | .690  (.07) |
| Battery not included | .567**  (.008) | .574  (.11) |
| **Maximum output wattage – <40w as a comparison group** | | |
| 40w≤output<85w | .406***  (<.001) | .406*  (.02) |
| Output≥85w | .555***  (<.001) | .556***  (.001) |
| Wattage output missing | .073  (.54) | .080  (.72) |
| **Pod kits (n=348)** | | |
| # of tanks/pods | .102**  (.01) | .099**  (.005) |
| Tank/pod volume size | .013  (.50) | .021  (.35) |
| **Battery capacity – <900mah as a comparison group** | | |
| 900mah≤capacity<1500mah | .161*  (.02) | .150***  (.001) |
| Capacity≥1500mah | .240*  (.02) | .222**  (.006) |
| Battery missing^a^ | .504***  (<.001) | .472**  (.02) |
| **Maximum output wattage – <40w as a comparison group** | | |
| 40w≤output<85w | .126  (.06) | .136*  (.05) |
| Output≥85w^a^ | -.013  (.82) | .040  (.86) |
| Wattage output missing | .046  (.45) | .041  (.45) |
| **Vape pens (n=41)** | | |
| Vape pen volume size | .034  (.09) | .037  (.12) |
| **Battery capacity – <900mah as a comparison group** | | |
| 900mah≤capacity<1500mah | .111  (.37) | .093  (.49) |
| Capacity≥1500mah | .135  (.41) | .119  (.36) |
| Battery missing | .750***  (<.001) | .765**  (.003) |
| **Maximum output wattage – <40w as a comparison group** | | |
| 40w≤output<85w | .000  (1.00) | -.001  (1.00) |
| Output≥85w | -.187  (.38) | -.244  (.50) |
| Wattage output missing | -.204  (.06) | -.193  (.12) |

Note: *** *P*<0.001. ** *P*<0.01. **P*<0.05. All regressions were controlled for accessories.

^a^: Since only 2 pod kits have output wattage greater than 85w and only 3 pod kits have missing battery capacity information, their estimated coefficients have limited statistical relevance.
